# How do private general practitioners manage tuberculosis cases? A survey in eight cities in Indonesia

**DOI:** 10.1186/s13104-015-1560-7

**Published:** 2015-10-14

**Authors:** Yodi Mahendradhata, Trisasi Lestari, Ari Probandari, Lucia Evi Indriarini, Erlina Burhan, Dyah Mustikawati, Adi Utarini

**Affiliations:** Center for Health Policy and Management, Faculty of Medicine, Gadjah Mada University, Sekip Utara, Yogyakarta, 55281 Indonesia; Department of Public Health, Faculty of Medicine, Gadjah Mada University, Sekip Utara, Yogyakarta, 55281 Indonesia; Department of Public Health, Faculty of Medicine, Sebelas Maret University, Jl. Ir. Sutami 36A, Surakarta, 57126 Indonesia; Department of Pulmonology and Respiratory Medicine, Faculty of Medicine, University of Indonesia, Persahabatan Hospital, Jl. Persahabatan Raya No. 1 Rawamangun, East Jakarta, 13230 Indonesia; National TB Control Pogramme, Directorate General for Disease Control and Environmental Health, Ministry of Health, Republic of Indonesia, Jalan Percetakan Negara No. 29 Gedung B Lantai 4, Central Jakarta, 10560 Indonesia

**Keywords:** Private practice, General practitioner, Guidelines, Indonesia, Tuberculosis

## Abstract

**Background:**

Private practitioners (PPs) in high-burden countries often provide substandard tuberculosis (TB) treatment, leading to increased risk of drug resistance and continued transmission. TB case management among PPs in Indonesia has not been investigated in recent years, despite longstanding recognition of inadequate care and substantial investment in several initiatives. This study aimed to assess case management practices of private general practitioners (GPs) in eight major cities across Indonesia.

**Methods:**

A cross-sectional survey of private GPs was carried out simultaneously in eight cities by trained researchers between August and December 2011. We aimed for a sample size of 627 in total, and took a simple random sample of GPs from the validated local registers of GPs. Informed consent was obtained from participants prior to interview. Diagnostic and treatment practices were evaluated based on compliance with national guidelines. Descriptive statistics are presented.

**Results:**

Of 608 eligible GPs invited to participate during the study period, 547 (89.9 %) consented and completed the interview. A low proportion of GPs (24.6–74.3 %) had heard of the International Standards for TB care (ISTC) and only 41.2–68.9 % of these GPs had participated in ISTC training. As few as 47.3 % (90 % CI: 37.6–57.0 %) of GPs reported having seen presumptive TB. The median number of cases of presumptive TB seen per month was low (0–5). The proportion of GPs who utilized smear microscopy for diagnosing presumptive adult TB ranged from 62.3 to 84.6 %. In all cities, a substantial proportion of GPs (12.0–45.5 %) prescribed second-line anti-TB drugs for treating new adult TB cases. In nearly all cities, less than half of GPs appointed a treatment observer (13.8–52.0 %).

**Conclusions:**

The pattern of TB case management practices among private GPs in Indonesia is still not in line with the guidelines, despite longstanding awareness of the issue and considerable trialing of various interventions.

## Background

The global burden of tuberculosis (TB) remains high even though it is a preventable and treatable disease. In 2012, there were an estimated 8.6 million new TB cases worldwide and 1.3 million TB-related deaths, including 320,000 among HIV-positive cases [1]. In the same year, there were an estimated 450,000 new cases of multidrug-resistant TB. Input from the private healthcare sector has long been advocated as essential for effective global TB control [2]. Generic regional and national strategies for a public–private mix (PPM) have been developed and endorsed since 2002 by the Stop TB Partnership Subgroup on PPM for TB Care and Control [3]. Engaging private providers in TB care and control through a PPM subsequently has been incorporated into the World Health Organization (WHO) stop TB strategy [4]. However, a large proportion of private care providers have still not engaged with national TB programs (NTPs) in many countries [5]. WHO thus proposed the involvement of private care providers as one of the key elements of the post-2015 global TB strategy and targets [6].

Indonesia ranks among the top five in the world for TB burden [1]. In 2012, 460,000 people in the country contracted TB, and there were 67,000 TB-related deaths [1]. A national survey on health-seeking behavior among presumptive TB cases showed that 54 % first sought treatment with a private practitioner (PP) [7], and many TB patients in Asian megacities prefer to seek care in the private sector [8]. Patients are either unaware of the free services available, perceive government services to be of poor quality, or are deterred by long waiting times and inconvenient consulting hours. Thus, private general practitioners (GPs) are preferred by many patients as they are more easily accessible than the public care providers, and charge lower consultation fees than private specialist physicians [9]. A survey conducted in 2004 in Jogjakarta, Indonesia reported inappropriate TB case management practices among PPs [10]. A recent study in Pakistan reported that PPs had less knowledge regarding the diagnosis and management of TB compared with physicians in the public sector [11]. Another recent study in India reported that TB diagnostic and treatment practices of most PPs were not in line with the International Standards for Tuberculosis Care (ISTC) [12]. These recent studies reaffirmed widespread consensus that treatment in the private-for-profit health sector is often substandard and unregulated, and can thus lead to drug resistance and continued transmission of TB [13].

The engagement of PPs with TB standards of care has been incorporated as one of the key components of Indonesia’s national strategic framework for TB control 2006–2010. Some PPM initiatives have also been initiated and systematically evaluated [14–16]. A national PPM has been a key component in the national strategy for TB control 2011–2014. However, there has been no systematic survey of TB case management practices among PPs since the previous survey in 2004 [10]. Such a survey is much needed to document the current situation, to provide a basis for evaluating current strategies, and to inform further policy discussions. For this purpose, this study assessed the case management practices of private GPs in eight major cities across Indonesia.

## Methods

### Study setting

The eight cities included in the study were provincial capitals and selected to represent the main geographical regions of Indonesia as follow: Java-Bali region (Jakarta, Bandung, Semarang, Surabaya); Sumatra region (Medan, Palembang); Kalimantan region (Banjarmasin); Eastern Indonesia region (Jayapura). The Java-Bali region, which encompasses over half of the country’s population, was represented with the largest number of cities (4). In each city, the primary care network consists of PPs (doctors, midwives, and nurses) and public community health centers, also staffed by doctors, midwives, and nurses. These first-line services are backed up by public and private hospitals which have been engaged in the directly observed treatment short-course (DOTS) program in Indonesia.

### Study design

A cross-sectional survey was carried out simultaneously in eight cities by trained researchers between August and December 2011. The study populations were all registered private GPs in the eight cities, including those who also worked in the public sector, and provided private healthcare. The cities were selected based on population size and number of private GPs, in consideration of the potential impact of TB interventions in these areas. The total population of the eight cities was 13,344,707, with 5837 private GPs, representing 10.5 % of the total number of nationally registered GPs in 2011. GPs in rural areas were not included because of their small number and their smaller potential contribution to TB control considering the limited resources of the NTP.

The absence of a national register of private GPs did not allow for the possibility of a nationally representative sample. We thus aimed for samples that were representative of the population of each city. The initial sampling frame was the register of private medical doctors in each city. These registers were then validated by cross-checking with databases of the local branches of the Indonesian Medical Association. For each city we calculated a minimum sample size to reach a precision of 10 % at the 90 % confidence level for an expected frequency of 50 % of private GPs complying with NTP guidelines. In total, we aimed for a sample size of 627 across all eight cities. A simple random sample of GPs was taken from the validated register. We excluded GPs who were retired, had taken specialist training, or who were unable to undergo the interview process for health reasons.

The questionnaire was developed in consultation with the NTP and the Indonesian Medical Association, and was pre-tested in interviews with GPs in a city not included in the study. Most questionnaire items (including those on diagnostic and treatment practices) were formulated in a multiple choice format to minimize potentially leading questions. Data collected included GP characteristics, whether presumptive TB cases had ever been seen in their practice, how many TB cases were seen in the last month, how the GP diagnosed and treated TB, and GP knowledge and perceptions of the ISTC. Presumptive TB was defined as an adult patient with a history of cough for 2 weeks or more, in line with NTP guidelines.

The survey was preceded by meetings with representatives of the local health authorities and local branches of the medical associations to coordinate the survey. If phone numbers were available, GPs were contacted to make an appointment for the researcher to visit. When a GP in the sample declined to be interviewed, was excluded, or was not located, the survey coordinators identified another GP randomly from the validated register. The coordinators were required to draw replacement samples repeatedly in order to reach the minimum sample size, within the limitation of the study duration and resources. Written informed consent was obtained prior to the interview, which lasted for about 15–30 min and was conducted in the Indonesian language.

Data were checked for completeness by the survey coordinator, and double data entry was performed. Diagnosis and treatment practices were evaluated based on compliance with the national guidelines for TB control [17]. Descriptive statistics were obtained using STATA software (Stata Corp., College Station, TX, USA). Confidence intervals (CIs) were determined for proportions and interquartile ranges (IQR) for medians.

Ethical approval was obtained from the ethics committee of the Faculty of Medicine, Gadjah Mada University. Permissions were obtained from the Ministry of the Interior. Endorsement was obtained from NTP and the Indonesia Medical Association.

## Results

We searched for a total of 1024 GPs from August to December 2011. A number (n = 273) could not be found (incorrect/incomplete address, moved, on leave, out of town, deceased). Of 751 GPs who were contactable, 143 were excluded (retired, ill, hearing loss, had taken specialist training), leaving 608 GPs, of whom 547 (89.9 %) consented to be interviewed. The basic characteristics of these GPs are presented in Table 1. In most cities, there were slightly more females than males. In all eight cities, the median duration of experience in offering private healthcare was more than 3 years. Many GPs were not aware of the ISTC (24.6–74.3 %), with less than half aware in five of the cities. Only 24.6 % (90 % CI: 15.9–33.3) of GPs in Medan were aware of the ISTC. Among those GPs who were aware of the ISTC, the proportion who considered it easy to apply in private practice ranged from 41.2 % (90 % CI: 19.7–62.6 %) in Medan to 68.9 % (90 % CI: 54.1–83.8 %) in Jayapura. Most GPs also had not undertaken ISTC training, with the proportion who had ISTC training ranging from 3.8 % (90 % CI: 0.7–10.9 %) in Medan to 41.5 % (90 % CI: 20.3–41.1 %) in Banjarmasin.

**Table 1:** Characteristics of interviewed private general practitioners (GPs) in eight cities in Indonesia (2011) and their knowledge and perceptions of the International Standards of Tuberculosis Care (ISTC)

City	Number of interviewed private GPs	Sex		Years of clinical practice			Aware of ISTC		Have participated in ISTC training		Perceived ISTC easy to apply in private practice	
		Male	Female	Median	Q1	Q3	n (%)^a^	90 % CI	n (%)^b^	90 % CI	n (%)^b^	90 % CI
Medan	69	30 (43.4 %)	39 (56.5 %)	6.0	3.0	12.0	17 (24.6)	15.9–33.3	2 (3.8)	0.7–10.9	7 (41.2)	19.7–62.6
Palembang	85	32 (37.6 %)	53 (62.4 %)	7.0	3.5	14.0	35 (41.2)	32.2–50.1	3 (5.7)	1.1–10.4	22 (62.9)	48.8–76.9
Banjarmasin	74	23 (31.1 %)	51 (68.9 %)	5.0	2.0	11.0	55 (74.3)	65.8–82.8	22 (41.5)	20.3–41.1	36 (65.5)	54.6–76.3
Jakarta	47	25 (53.2 %)	22 (46.8 %)	4.0	2.0	10.0	21 (44.7)	32.4–56.9	1 (8.9)	0.2–13.9	9 (42.9)	23.8–61.9
Bandung	52	20 (38.5 %)	32 (61.5 %)	6.0	2.0	11.0	22 (42.3)	30.7–53.9	1 (8.9)	0.2–12.6	13 (59.1)	40.6–77.6
Semarang	90	40 (44.4 %)	50 (55.6 %)	10.0	5.0	17.0	53 (58.9)	50.2–67.6	8 (15.1)	4.5–16.8	29 (54.7)	43.2–66.3
Surabaya	86	43 (50.0 %)	43 (50.0 %)	7.0	4.0	12.0	33 (38.4)	29.6–47.1	3 (5.7)	1.1–10.3	14 (42.4)	27.6–57.2
Jayapura	44	18 (40.9 %)	26 (59.1 %)	6.0	2.0	12.0	29 (65.9)	53.8–78.1	13 (24.5)	17.8–44.7	20 (68.9)	54.1–83.8

*CI* confidence interval

^a^Denominator: number of private GPs interviewed

^b^Denominator: number of private GPs who have heard of the ISTC

Many GPs had never seen presumptive TB. The proportion of GPs who reported that they had seen presumptive TB ranged from only 47.3 % (90 % CI: 37.6–57.0 %) in Banjarmasin to 88.6 % (90 % CI: 80.5–96.8 %) in Jayapura (Table 2). The median number of presumptive TB cases seen per month was relatively low, with the highest median only 5 (IQR: 2–10) in Semarang. Many GPs did not utilize smear microscopy for diagnosing adult presumptive TB, with the proportion ranging from 62.3 % (90 % CI: 51.0–73.5 %) in Surabaya to 84.6 % (90 % CI: 74.7–94.5 %) in Jayapura.

**Table 2:** Number of presumptive tuberculosis cases seen by private GPs in eight cities in Indonesia (2011) and utilization of smear microscopy in the diagnostic process

City	Ever seen presumptive TB in the past year		Presumptive TB per month			Utilized smear microscopy in diagnosing TB	
	n (%)^a^	90 % CI	Median	Q1	Q3	n (%)^b^	90 % CI
Medan	47 (68.1)	58.7–77.5	4.0	0.0	15.0	36 (76.6)	66.1–87.1
Palembang	59 (69.4)	61.1–77.8	3.0	0.0	10.0	37 (62.7)	52.1–73.3
Banjarmasin	35 (47.3)	37.6–57.0	0.0	0.0	2.0	23 (65.7)	51.9–79.5
Jakarta	32 (68.1)	56.5–79.6	2.0	0.0	7.0	22 (68.8)	54.6–82.9
Bandung	27 (51.9)	40.2–63.6	1.0	0.0	3.5	20 (74.1)	59.4–88.7
Semarang	75 (83.3)	76.8–89.9	5.0	2.0	10.0	50 (66.7)	57.5–75.8
Surabaya	53 (61.6)	52.8–70.4	2.0	0.0	5.0	33 (62.3)	51.0–73.5
Jayapura	39 (88.6)	80.5–96.8	2.5	1.0	5.0	33 (84.6)	74.7–94.5

*TB* tuberculosis

^a^Denominator: number of interviewed private GPs (see Table 1)

^b^Denominator: number of private GPs ever seen presumptive TB

The proportion of GPs who had treated a TB case in the past year was relatively low, and less than half in most cities, ranging from only 18.9 % (90 % CI: 11.4–26.4 %) in Banjarmasin to 54.5 % (90 % CI: 42.2–66.9 %) in Jayapura (Table 3). Among GPs who had treated a TB case, many did not prescribe a regimen in line with the NTP guidelines. The proportion prescribing in line with the guidelines ranged from 52.5 % (90 % CI: 39.0–65.9 %) in Semarang to 87.1 % (90 % CI: 76.7–97.5 %) in Jakarta. Notably, in all cities there was a considerable proportion of GPs who prescribed second-line anti-TB drugs for treating new adult TB cases, reaching as high as 45.5 % (90 % CI: 30.5–60.4 %) in Medan. Most GPs in all cities did not prescribe regimens in the form of a fixed dose combination (FDC). In Jakarta, none of the GPs prescribed a FDC. The highest proportion of GPs prescribing a FDC was in Banjarmasin, at only 35.7 % (90 % CI: 12.2–59.2 %). In nearly all cities, the majority of GPs did not appoint a treatment observer to ensure treatment compliance, ranging from only 13.8 % (90 % CI: 2.7–24.9 %) in Surabaya to 52.0 % (90 % CI: 34.6–69.4 %) in Bandung. In all cities, the majority of GPs did not report TB cases to the NTP, ranging from only 12.5 % (90 % CI: 0.7–24.3 %) in Jayapura to 42.5 % (90 % CI: 29.2–55.8 %) in Semarang.

**Table 3:** TB case management practices of private GPs in eight cities in Indonesia, 2011

City	Ever treated a TB case in the past year		Prescribed regimen according to NTP guideline		Prescribed second-line drugs		Appointed treatment observer		Reported TB case to NTP	
	n (%)^a^	90 % CI	n (%)^b^	90 % CI	n (%)^b^	90 % CI	n (%)^b^	90 % CI	n (%)^b^	90 % CI
Medan	33 (47.8)	37.9–57.7	24 (72.7)	59.4–86.1	15 (45.5)	30.5–60.4	14 (42.4)	27.6–57.2	7 (21.2)	8.9–33.5
Palembang	29 (34.1)	25.6–42.6	22 (75.9)	62.1–89.6	6 (20.7)	7.7–33.7	13 (44.8)	28.8–60.8	8 (27.6)	13.2–41.9
Banjarmasin	14 (18.9)	11.4–26.4	10 (71.4)	49.2–93.6	4 (28.6)	6.4–50.8	5 (35.7)	12.2–59.2	4 (28.6)	6.3–50.8
Jakarta	31 (66.0)	54.6–77.7	27 (87.1)	76.7–97.5	4 (12.9)	2.5–23.3	7 (22.6)	9.6–35.5	9 (29.0)	14.9–43.1
Bandung	25 (48.1)	36.6–59.5	21 (84.0)	71.2–96.8	3 (12.0)	0.6–23.3	13 (52.0)	34.6–69.4	10 (40.0)	22.9–57.1
Semarang	40 (44.4)	35.8–53.1	21 (52.5)	39.0–65.9	7 (17.5)	7.2–27.8	10 (25.0)	13.3–36.7	17 (42.5)	29.2–55.8
Surabaya	29 (33.7)	25.3–42.1	19 (65.5)	50.2–80.7	8 (27.6)	13.2–41.9	4 (13.8)	2.7–24.9	8 (27.6)	13.2–41.9
Jayapura	24 (54.5)	42.2–66.9	19 (79.2)	64.6–93.7	3 (12.5)	0.6–24.3	9 (37.5)	20.2–54.8	3 (12.5)	0.7–24.3

*NTP* National Tuberculosis control Program

^a^Denominator: number of interviewed private GPs (see Table 1)

^b^Denominator: number of GPs who ever treated TB cases

## Discussion

Our results show that private GPs in Indonesia do see presumptive TB, although the number per GP is notably low. The data also suggest that TB case management practices of private GPs are generally not in line with NTP guidelines.

Many private GPs were not aware of the ISTC, and the majority of GPs have not undertaken ISTC training. The lack of knowledge of PPs regarding current TB management has been documented in other settings. A study in Oman found that private GPs had significantly lower knowledge of TB compared with public GPs [18]. According to a study in India, doctors in the public sector had 2.1 times better knowledge of TB management than private sector doctors [19]. A recent study in Ethiopia found that a significant proportion of PPs did not have satisfactory knowledge about TB case management [20]. A scoping review of 34 studies revealed that private care providers lacked comprehensive knowledge of national treatment guidelines [21]. The observed pattern of limited knowledge on TB management has been frequently attributed to lack of training in medical schools and limited efforts for continuing medical education.

In the context of our study population, the ISTC is yet to be integrated effectively into the medical curriculum since its introduction in 2006. However, the Indonesian Medical Association has been coordinating a campaign that includes: dissemination of information on the ISTC via Indonesian medical journals; inclusion of the ISTC in presentations at professional society meetings; and utilization of the ISTC as the basis for continuing medical education programs [22]. In more recent years, efforts to disseminate the ISTC in Indonesia have been intensified. The Indonesian Medical Association has conducted workshops on ISTC in all 33 provinces [23]. The ISTC has also been incorporated in the NTP guidelines and materials for national training in Indonesia. Between 2010 and 2013, 97 pulmonologists and 61 hospitals in three provinces have been using the ISTC [3]. These efforts evidently need to be redoubled, monitored, and evaluated to ensure broader knowledge of the ISTC among private GPs. Further studies should also evaluate whether the knowledge and practices of those exposed to the ISTC differ from those with no exposure.

Not all private GPs have seen presumptive TB, and the volume of presumptive TB seen per month is low. This is in line with findings from a previous survey conducted among PPs in Jogjakarta, Indonesia [10]. In contrast, a recent impact evaluation of private sector engagement to increase TB case detection in Karachi, Pakistan, reported a doubling in case notifications [8]. Case notification results obtained across different projects that involve PPs in TB control vary widely. A review of six in India showed that the contribution of these projects to case notification varied between 2 and 26 % [24]. The ratio of new cases notified per year by PPs documented in this review varied between 1.4 and 18.8. A more recent study evaluating PPM scaling up in 14 cities in India also revealed large variations between cities in case notifications [25]. These observed variations evidently highlight the influence of different intervention designs and different contexts (e.g., epidemiology, health system). Nevertheless, our findings indicate that even high-level engagement of private GPs in high-burden settings would not necessarily increase case notification substantially. We need to always consider the local context [26].

There was a considerable proportion of private GPs who did not utilize smear microscopy for diagnosing TB, even though sputum smear microscopy services are widely accessible in public and private facilities in all eight cities. This also reaffirms findings from a previous survey conducted among PPs in Jogjakarta, Indonesia [10]. Studies carried out in the Philippines [27] and Kenya [28] similarly reported that PPs relied more on a chest X-ray than sputum microscopy. More recently, a study among PPs in India reported low compliance to the ISTC diagnosis standard, implying that only a few rightly suspected TB and performed sputum microscopy for diagnosis of TB as their first choice [12]. Such substandard diagnostic practice indicates an over reliance of PPs to adopt investigations that give immediate results, and the pressure on them to provide faster results for their clients. This observed pattern also raises concerns in regard to the use of inappropriate and suboptimal TB diagnostic tests (e.g., current serological tests) in the private sector [29, 30].

The case management pattern is the most disturbing of all our findings. Many private GPs in our study did not prescribe a drug regimen in line with NTP guidelines, did not prescribe FDC, did not appoint a treatment observer, and did not report a TB case to the NTP. This pattern was also documented in a previous survey conducted among PPs in Jogjakarta, Indonesia [10]. Similar findings have also been reported in other settings. A study in Mumbai, India, found that the vast majority of PPs were unable to provide a correct prescription for treating TB [31]. A more recent study in Andhra Pradesh, India, reported that only one-third of PPs prescribed the correct treatment regimen for a new TB case with no previous history of TB treatment, and two-thirds prescribed a large variety of different prescriptions in terms of drugs, dosage and duration [12]. They also reported similar perplexing findings that many PPs prescribed second-line anti-TB drugs, such as fluoroquinolones, against the recommendations in the ISTC and any other TB treatment guidelines. The widespread lack of use of non-FDC first-line TB drugs and prescribing of second-line anti-TB drugs for new cases in the private sector in Indonesia has also been documented in another recent study [32]. Further studies should investigate more closely the factors leading to these worrisome practices. Poor TB case management practices are known to increase the risk of anti-TB drug resistance [33]. Thus, our findings of inappropriate case management among private GPs in these eight cities should be of considerable concern to the NTP. However, the small number of presumptive TB cases treated by private GPs suggests that the costs of intervention and the potential impact toward prevention of multidrug-resistant TB should be cautiously considered beforehand.

We missed our target sample size in several cities, which limits the power of our study in those cities and the possibility of conducting more subtle analysis. This was mostly related to the failure to find many of the sampled GPs. However, the response rate is high and the similar case management patterns observed in all cities suggests that it is unlikely that a larger sample size would change the pattern substantially. Because of the limited sample size, we did not exclude private GPs who also worked in government health facilities. Dual practice is widespread in Indonesia [34], so it would in any case be very difficult to generate relevant and meaningful assessment of knowledge and practices of purely private GPs. The private GPs were asked to estimate the number of presumptive TB seen in retrospect. This could have been more accurately measured through a prospective registration, but was not possible with the resources available. The low number of presumptive TB seen by private GPs, however, suggests that it is unlikely that they had exaggerated the numbers. The reverse could also be true; i.e., the GPs may have underplayed the number of presumptive TB cases they see as they were aware that the study was assessing their knowledge and prescribing practices. However, consistency with a previous study [10] suggests that the former is more probable than the latter. We also asked how GPs diagnosed and treated TB cases in general rather than specifically per case, in line with our aim of assessing the pattern of average practice/behavior of GPs. Direct observations would have been more accurate, but was not possible. The documented general pattern of poor compliance to NTP guidelines, however, suggests that it is unlikely that interviewed GPs have overstated their compliance. In addition, we highlighted the assessment of GP awareness of the ISTC rather than awareness of NTP guidelines. Thus, even though ISTC and NTP guidelines are related, the negative finding on awareness would be more suggestive of low effectiveness of ISTC dissemination efforts, rather than lack of knowledge of NTP guidelines.

## Conclusions

Our study revealed that the pattern of TB case management practices among private GPs in Indonesia in 2011 were still not markedly different from the disturbing pattern of inappropriate practices documented in the 2004 PP survey [10], despite longstanding recognition of the issue and considerable trialing of various PPM schemes [14–16]. There is an urgent need to rethink current PPM strategies and consider other potential approaches. For example, regulations have long been applied as a potential response to poor quality health care and have now evolved beyond costly conventional forms of command and control [35]. This should be borne in mind along with other innovative strategies when considering the issue of poor quality care among PPs, particularly while formulating upcoming national and global strategies for TB control.
